# Patient, carer and family experiences of seeking redress and reconciliation following a life‐changing event: Systematic review of qualitative evidence

**DOI:** 10.1111/hex.13820

**Published:** 2023-07-14

**Authors:** Liz Shaw, Hassanat M. Lawal, Simon Briscoe, Ruth Garside, Jo Thompson Coon, Morwenna Rogers, G. J. Melendez‐Torres

**Affiliations:** ^1^ Exeter Policy Research Programme Evidence Review Facility, Faculty of Health and Life Sciences, St Luke's campus University of Exeter Exeter UK

**Keywords:** complaint, fair process, framework synthesis, harm, NHS, patient safety

## Abstract

**Introduction:**

We conducted a systematic review of qualitative evidence to improve understanding of the processes and outcomes of redress and reconciliation following a life‐changing event from the perspectives of individuals experiencing the event and their families.

**Methods:**

We searched six bibliographic databases for primary qualitative evidence exploring the views of individuals who have experienced a life‐changing event, and/or their family or carers, of redress or reconciliation processes. This was supplemented with targeted database searches, forward and backward citation chasing and searches of Google Scholar and relevant websites. Title and abstract and full‐text screening were undertaken independently by two reviewers. Data extraction and quality appraisal were conducted by one reviewer and checked by a second. We used a best‐fit framework synthesis approach, drawing upon procedural and restorative justice concepts.

**Findings:**

Fifty‐three studies (61 papers) were eligible for inclusion. Forty‐one studies (47 papers) were included in the synthesis, from which we identified four themes. Three themes ‘Transparency’, ‘Person‐centered’ and ‘Trustworthy’ represent the procedural elements required to support a fair and objective process. The fourth, ‘Restorative justice’ encapsulates how a fair process feels to those who have experienced a life‐changing event. This theme highlights the importance of an empathic relationship between the different parties involved in the redress‐reconciliation process and the significance of being able to engage in meaningful action.

**Conclusion:**

Our findings highlight the procedural aspects and context of redress‐reconciliation processes required to ensure that the process and outcomes are experienced as fair. These criteria may be applied to the processes used to investigate both recent and historical patient safety events.

**Public Contribution:**

One member of the public affiliated with the Exeter Policy Research Programme Evidence Review Facility helped develop the review protocol. Two people with experience of medically life‐changing events provided insight which corroborated our findings and identified important limitations of the evidence included in this review.

## 1 BACKGROUND

Following adverse medical events resulting in death or life‐changing disability, investigations or inquiries are often conducted to establish what has happened, who was responsible and to identify opportunities for health organisations to improve care and achieve resolution for families. Efforts across different healthcare systems across the globe to improve patient safety and incident reporting include increasing surveillance of adverse events and refining payment incentives to reduce their occurrence^1^ and improving guidelines for reporting adverse events and associated learning systems.^2^

Within the United Kingdom, the system for managing complaints against the National Health Service (NHS), the ‘NHS Litigation Authority’ was reorganised and rebranded ‘NHS Resolution’ in 2017, with the intention to encourage an early settlement of cases and increased learning from past mistakes and prevention of future errors.^3^ Additional efforts to change the culture around patient safety issues and complaints include the publication of the NHS Patient Safety Strategy (2019), which provides a long‐term plan for the NHS to continuously improve the safety and culture of systems.^4^ The ‘Medical Examiner’ role was announced in 2020, a position which aims to provide vital insight into deaths following problems in care and inform future improvements in safety and act as a resource for bereaved families.^5^

However, recent policy and system changes do not address long‐term unresolved cases. Members of the UK Parliament have received calls to establish a process that addresses the concerns of patients, families and staff seeking justice following adverse events that occurred 20 years ago or more.^6^, ^7^ These cases have often been subject to multiple reviews, but some families feel that justice has not yet been achieved and remain traumatised and angry. We wished to better understand how health services can improve how they support and respond to those seeking justice following an adverse event, particularly those whose cases are historic (non‐recent).

There is little to no primary or secondary evidence examining patient or family/carer experiences of seeking justice for a historical serious adverse event within a healthcare setting. However, a small body of primary qualitative evidence across the international literature explored the experiences of patients and/or their families of redress and reconciliation processes for recent patient safety events within the healthcare and criminal justice fields. In this paper, we summarise the main findings of a systematic review of qualitative research commissioned by the UK Department of Health and Social Care (DHSC) to draw on international literature to improve the understanding of the processes and outcomes of redress and reconciliation following a life‐changing event from the perspectives of individuals experiencing the event and their families.^8^ Our research questions were:

1. What aspects of the processes and outcomes of redress and reconciliation following a life‐changing event lead the individual and/or family to feel that they were/were not treated fairly and appropriately?
2. How do these perceptions vary over time following the initial event?

## 2 METHODS

Our protocol was registered on PROSPERO.^9^ The methods used to conduct and report this review were consistent with best practice guidelines for conducting systematic reviews and reporting qualitative evidence synthesis.^10^, ^11^, ^12^ Members of the public and representatives of DHSC provided their input throughout the review process (see Appendix A).

### 2.1 Identification of evidence

We combined bibliographic databases, forward citation and web searching and checking reference lists. Searches spanned health and social care and criminal justice to draw on a breadth of research on justice‐seeking. Search terms (Appendix B) were derived from the titles, abstracts and indexing terms (e.g., MeSH in MEDLINE) of articles identified via scoping searches. We used a qualitative study‐type filter with additional search terms based on our preidentified set of journal articles.^13^ The final search was translated for use in the following six bibliographic databases on 25 February 2022: ASSIA (via ProQuest), MEDLINE (via Ovid), CINAHL (via EBSCO), HMIC (via Ovid), Social Science Citation Index (via Web of Science) and International Bibliography of the Social Science (IBSS) (via ProQuest). We carried out two additional searches using the IBBS (ProQuest) bibliographic database to search for studies on family members' experiences of inquests or negligence.

Included primary studies and systematic reviews of interest identified by bibliographic database searches were source studies for forward citation searching using the Science Citation Index and Social Sciences Citation Index (via Web of Science). We manually inspected the reference list of all included studies. Web searches were carried out using the Google Scholar search engine and relevant websites. We also searched the HeinOnline database.

We applied the inclusion criteria (Appendix C) to the title and abstracts of 100 bibliographic database search results to ensure consistent application of criteria. Two reviewers undertook title and abstract screening of each citation independently (L.S. and H.L.), with disagreements resolved through discussion or referral to a third reviewer as required (G.J. M.T.). Full‐texts of each paper were assessed in the same way.

### 2.2 Data extraction and quality appraisal

Two reviewers extracted summary data for all included studies (L.S. and H.L.). These data included the method of study identification, first author, date, title, aim, sector/field, country, year data collected, stage of justice‐seeking process represented, participants' relevant experience, participants providing views, data collection method and quantity of first‐/second‐order construct data relevant to our research questions.

We then grouped included studies according to the type of life‐changing event experienced. Types of events include medical, sexual abuse, suicide, occupational, homicide, death in custody/police killing and missing persons. Each of these groups was further subdivided to reflect the stage of the justice or redress‐reconciliation process, which was the focus of each paper.

Studies with the highest quantity of data relevant to our research questions were prioritised for full data extraction, quality appraisal and framework synthesis to manage the higher‐than‐anticipated number of studies eligible for inclusion. This was undertaken by one reviewer (L.S.) and checked by a second (H.L.). This deviation from our protocol was approved by all review stakeholders. Prioritised studies included all those from the medical field and a selection of studies from nonmedical fields identified via purposive sampling to support subthemes where data from the medical field were Iimited.

Extraction of the descriptive data for prioritised studies was undertaken by one reviewer (L.S., H.L.) and checked by a second (L.S., H.L., G.J.M.T., S.B., R.G., J.T.C.) using Microsoft Excel, with disagreements settled by a third reviewer if necessary. We used the Wallace^14^ checklist to appraise the quality of these studies using the same process. Details of data extracted can be viewed in the full report.^8^ One reviewer extracted the first‐ and second‐order constructs from the results and discussion sections into a framework (L.S.), which was then checked by a second reviewer (H.L.).

### 2.3 Best‐fit framework synthesis

Our initial best‐fit framework was based on concepts derived from the accountability for reasonableness literature,^15^, ^16^, ^17^ which proposes four conditions for priority setting within healthcare to be considered fair and legitimate: (1) publicity, (2) relevance, (3) appeals and (4) enforcement.^15^, ^16^, ^17^ This framework was shared with other team members for discussion and revision while two researchers (L.S. and H.L.) independently piloted the framework on two of the four prioritised medical studies. The subsequent framework synthesis process and details regarding how the framework was developed are provided in Appendices D and E.

## 3 FINDINGS

**Figure 1:**
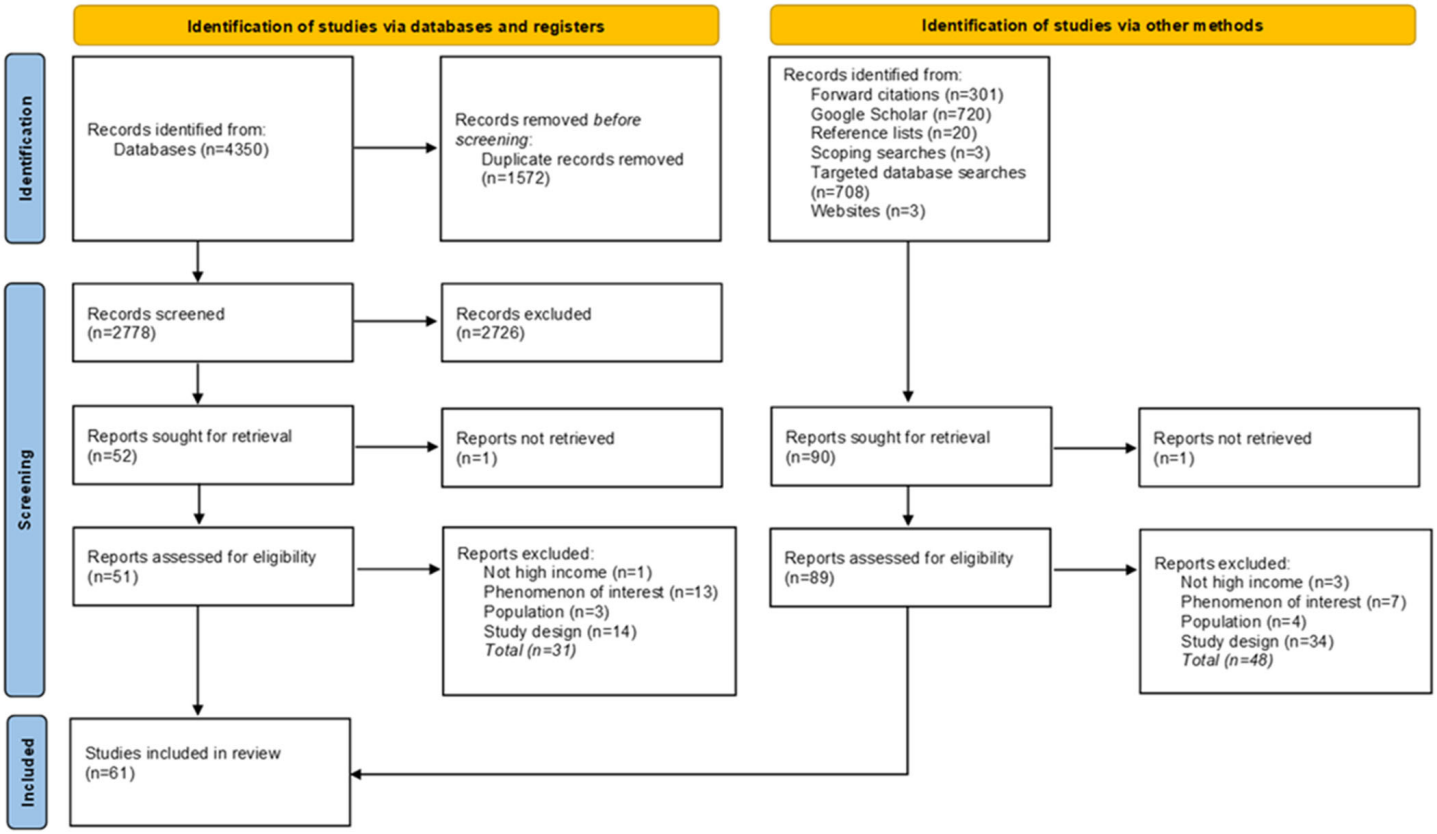
PRISMA diagram.

Fifty‐three studies (represented by 61 articles) were eligible for inclusion in this review (see Figure 1).^18^, ^19^, ^20^, ^21^, ^22^, ^23^, ^24^, ^25^, ^26^, ^27^, ^28^, ^29^, ^30^, ^31^, ^32^, ^33^, ^34^, ^35^, ^36^, ^37^, ^38^, ^39^, ^40^, ^41^, ^42^, ^43^, ^44^, ^45^, ^46^, ^47^, ^48^, ^49^, ^50^, ^51^, ^52^, ^53^, ^54^, ^55^, ^56^, ^57^, ^58^, ^59^, ^60^, ^61^, ^62^, ^63^, ^64^, ^65^, ^66^, ^67^, ^68^, ^69^, ^70^, ^71^, ^72^, ^73^, ^74^, ^75^, ^76^, ^77^, ^78^

### 3.1 Study characteristics

Details of the study and participant characteristics are provided elsewhere.^8^

Forty‐one studies (represented by 47 articles) were prioritised for framework synthesis.^18^, ^19^, ^20^, ^22^, ^23^, ^25^, ^27^, ^29^, ^30^, ^31^, ^32^, ^34^, ^35^, ^36^, ^38^, ^39^, ^42^, ^43^, ^44^, ^47^, ^48^, ^49^, ^50^, ^51^, ^52^, ^53^, ^54^, ^55^, ^56^, ^57^, ^58^, ^59^, ^60^, ^61^, ^63^, ^64^, ^65^, ^68^, ^69^, ^70^, ^71^, ^72^, ^73^, ^74^, ^75^, ^76^, ^77^, ^78^ The most common countries where studies were conducted included the United States of America (*n* = 12),^27^, ^30^, ^31^, ^38^, ^39^, ^43^, ^51^, ^52^, ^58^, ^59^, ^64^, ^68^, ^71^, ^72^ Australia (*n* = 8)^19^, ^22^, ^29^, ^34^, ^35^, ^36^, ^41^, ^54^, ^61^, ^65^, ^69^, ^74^ and the United Kingdom (*n* = 9).^18^, ^20^, ^25^, ^48^, ^55^, ^57^, ^76^, ^77^, ^78^ Adverse events occurred within following fields: medical (*n* = 31),^19^, ^23^, ^31^, ^32^, ^34^, ^35^, ^36^, ^43^, ^44^, ^47^, ^48^, ^49^, ^50^, ^51^, ^52^, ^53^, ^54^, ^55^, ^56^, ^57^, ^58^, ^59^, ^60^, ^63^, ^65^, ^68^, ^69^, ^70^, ^71^, ^72^, ^74^, ^75^, ^76^, ^77^, ^78^ homicide (*n* = 3),^27^, ^30^, ^38^, ^39^ sexual abuse (*n* = 2),^22^, ^29^, ^64^ employment/work‐related death (*n* = 2),^42^, ^61^ death in custody (*n* = 1)^25^ and suicide (*n* = 2).^18^, ^20^ The majority of medical studies focused on the disclosure phase of the justice‐seeking process (*n* = 17).^19^, ^32^, ^34^, ^35^, ^36^, ^43^, ^48^, ^51^, ^53^, ^54^, ^56^, ^57^, ^58^, ^65^, ^68^, ^69^, ^70^, ^71^, ^72^, ^74^, ^75^

Most studies scored positively on at least 8 of the 14 Wallace checklist items (range 2–13^52^),^27^, ^29^, ^32^, ^53^, ^70^ aside from four studies which scored positively in 2, 5 and 6 out of 14 items, respectively.^25^, ^52^, ^55^, ^78^ See Appendix F for scores for each prioritised article on individual quality appraisal items.

## 4 FRAMEWORK SYNTHESIS

The synthesis identified four main themes: (1) The need for Transparency, (2) Person‐centredness, (3) Trustworthy and (4) Restorative Justice. The subthemes contained within these themes, and supporting papers, are summarised in Appendix G.

**Figure 2:**
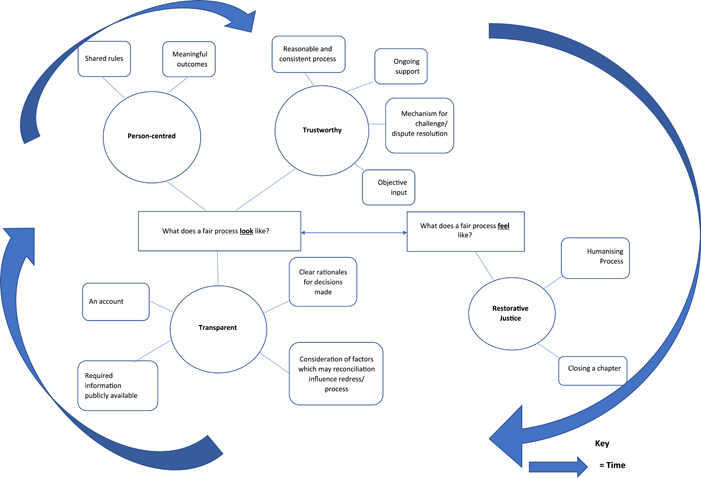
Relationship between themes.

The first three themes explore what a fair process looks like according to justice‐seekers. The final theme explores what a fair process feels like. The relationship between these four themes, and their subthemes, is shown below in Figure 2.

### 4.1 What does a fair process look like?

#### 4.1.1 Theme 1: Transparency

People seeking redress and reconciliation following a life‐changing event need a comprehensive account of the harm they have experienced, the circumstances leading up to it and what is being done to ensure it does not happen again.^20^, ^25^, ^27^, ^32^, ^34^, ^35^, ^36^, ^38^, ^39^, ^42^, ^43^, ^44^, ^47^, ^49^, ^50^, ^51^, ^52^, ^53^, ^54^, ^55^, ^58^, ^59^, ^60^, ^61^, ^65^, ^68^, ^69^, ^70^, ^71^, ^72^, ^74^, ^75^, ^76^, ^77^, ^78^

I needed to find out everything that went on, how it went on, how they was [sic] able to prosecute or catch him…The more I knew about what was going on…the better off I was. (Oklahoma City bombing, Madeira et al.^39^ ^, p.1500^)

Being able to meet to discuss what happened with those directly involved may enhance the perceived trustworthiness of the account received.

Perceived transparency of, and trust within, the redress‐reconciliation process can be enhanced if it is easy to access information about the event, how to access the redress‐reconciliation process itself and justice‐seekers' rights within it. The extent to which justice‐seekers are signposted to the information and support they need can also support this.^18^, ^22^, ^25^, ^27^, ^29^, ^32^, ^34^, ^35^, ^36^, ^39^, ^42^, ^43^, ^44^, ^47^, ^48^, ^49^, ^50^, ^51^, ^53^, ^54^, ^55^, ^56^, ^57^, ^59^, ^60^, ^61^, ^63^, ^65^, ^68^, ^70^, ^71^, ^75^, ^76^, ^77^, ^78^ Any perception of avoidance, dismissal or bias in favour of medical professionals, or that professionals are not being open and transparent, can lead to feelings of anger, uncertainty and suspicion on the part of the people seeking redress‐reconciliation and the perception that their needs and views are not valued.

They just seem to want to fob us all off and hope we'll go away. They don't seem to be taking the complaint serious enough and being proactive about doing something about it. They just seem to be wanting to avoid the issue completely, and thinking, ‘Well, not many women complain’. (Patient—Complained about treatment following painful invasive investigation, Martin^57^ ^, p.4^)

Other systemic factors may influence decision‐making within, and thus the perceived transparency and fairness of, the justice‐seeking process.^23^, ^25^, ^27^, ^29^, ^30^, ^42^, ^55^, ^56^, ^57^, ^59^, ^60^, ^70^, ^75^, ^76^, ^77^, ^78^ The availability of funding for both individuals seeking justice and those awarding compensation is one influential factor.

…you only get Legal Aid for a certain amount of money and once I'd been to that lawyer and they'd said ‘no’, that was the money used up and I would have to pay, which would go to tens of thousands of pounds. (Bereaved female relative, Melville et al.^77^ ^, p.34^)

Another is how easily the redress‐reconciliation process can record and respond to individual concerns and experiences of justice‐seekers.

…we didn't really have the chance to complain down the normal route because it was superseded by this investigation…although we have had our input and communicated our feelings and our experience—a little bit like one‐way traffic. (Parent of child detained for over 24 h under the Mental Health Act, Martin et al.,^57^ ^,p.5^)

These factors should be explicitly discussed between all stakeholders to maintain transparency.

People seeking justice appreciate being provided with a clear rationale for the decisions made during the process.^22^, ^29^, ^30^, ^38^, ^39^, ^49^, ^55^, ^63^, ^77^ Despite attempts made by professionals and justice‐seekers themselves to provide/obtain clarity, the reasons behind decisions made during the justice‐seeking process are not always transparent. This contributes to perceptions of bias and lack of consistency in the justice‐seeking process, leaving patients and their families feeling unheard.

#### 4.1.2 Theme 2: Person‐centred

Patients and their families desired the reconciliation process to be based on a shared understanding of what had occurred, their needs as justice‐seekers and consideration of what they wanted to achieve.^18^, ^22^, ^25^, ^27^, ^29^, ^30^, ^31^, ^32^, ^35^, ^36^, ^48^, ^49^, ^50^, ^51^, ^52^, ^54^, ^55^, ^57^, ^58^, ^59^, ^60^, ^65^, ^70^, ^75^, ^76^, ^77^

I loved that [the hospital] asked me ‘How can we address this for you?’. (Participant, Moore et al.^60^ ^, p.792^)

The process should consider whether the timing, method of involvement and location are convenient and have a clearly defined end‐point which all stakeholders agree upon. Considering the extent to which people seeking redress‐reconciliation wish to be involved may increase perceptions of transparency and trust in the process and in turn the perception of fairer outcomes.

If the idea is to learn, how much is lost in the learning when people are pushed at times when they actually can't mentally or physically do any more other than survive what has happened? (Relative of child detained for over 24 h under Mental Health Act—Event review, Martin et al.^57^ ^, p.6^)

The outcomes sought through redress‐reconciliation processes varied.^18^, ^20^, ^25^, ^27^, ^29^, ^30^, ^31^, ^32^, ^34^, ^35^, ^36^, ^38^, ^39^, ^42^, ^43^, ^44^, ^47^, ^48^, ^50^, ^51^, ^52^, ^53^, ^54^, ^55^, ^56^, ^57^, ^58^, ^59^, ^60^, ^61^, ^63^, ^64^, ^65^, ^68^, ^69^, ^70^, ^71^, ^72^, ^74^, ^75^, ^76^, ^77^, ^78^ Key outcomes included receiving answers to their questions and receipt of an apology, incorporating expressions of remorse and an admission of responsibility, accompanied by actions to assure them that the harm would not happen again and that future health and financial needs will be met.

The apology …in one sense was very short. It meant so much. I was amazed at how my feelings could change. (Participant, Hovey et al.^75^ ^, p.270^)

Other concepts include the desire for appropriate sanctions against those perceived to be responsible, and the need to ensure that the final verdict reflects the evidence provided and incorporates the views of those harmed. Outcomes sought were individual to the justice‐seeker and influenced by responses received from institutions or individuals perceived to have done them harm.

I just feel that the medical profession is so scared of being sued that it closes down…if they listened to people, and tried to rectify the mistakes, in a way that people actually wanted, there would be less compensation and it's less confrontational. (Participant life‐changing event review, McQueen et al.^76^ ^, p.8^)

This indicates that justice‐seekers need to be consulted at the early stages of the process regarding how they can be supported to close this chapter of their lives. The processes needed to achieve this require ongoing rapport between the different stakeholders throughout the duration of the redress‐reconciliation pathway.

#### 4.1.3 Theme 3: Trustworthy

Justice‐seekers need to be supported to access and maintain engagement with a preplanned, consistent process that centres around their needs.^18^, ^22^, ^25^, ^27^, ^29^, ^30^, ^31^, ^32^, ^34^, ^35^, ^36^, ^42^, ^44^, ^47^, ^48^, ^49^, ^50^, ^51^, ^52^, ^53^, ^54^, ^55^, ^56^, ^57^, ^58^, ^59^, ^60^, ^61^, ^65^, ^68^, ^74^, ^75^, ^76^, ^77^ Trust in the redress‐reconciliation process may be enhanced by using a formal pathway which promotes a two‐way dialogue between professional stakeholders and those who have experienced harm.

I was visited once by the obstetrician when I was in hospital…she came to visit me then, but it wasn't a planned [meeting]… so I didn't have any questions planned or anything. (Patient, Iedema et al.^36^ ^, p.94^)

Justice‐seekers appreciated their experiences and views being actively sought to inform the process and outcomes, as well as the involvement of the individuals they perceived as responsible for the harm.

I wanted mediation with the doctor who was responsible…I still can't get closure because I haven't yet spoken to that doctor. (Patient, Moore et al.^59^ ^, p.793^)

People wanted the process to be conducted in a timely manner, to limit the negative emotional impact of ongoing uncertainty, grief and fatigue associated with a lengthy, ongoing investigation.

We are drawing this [the review] out longer and longer and longer. And I have to be careful, I don't drown myself in this whole process…I shouldn't have to sacrifice my own health and wellbeing just to get answers. (Participant, McQueen et al.^76^ ^, p.6^)

A process which does not fully account for the needs, views and experiences of harmed individuals may be met with formal challenges, as they seek to have their perceptions accurately reflected in formal accounts of the life‐changing event and represented in the final outcome of the redress‐reconciliation process.

Justice‐seekers require ongoing financial and emotional support provided by advocates and/or family members to access and maintain their involvement.^25^, ^29^, ^30^, ^32^, ^35^, ^36^, ^38^, ^42^, ^43^, ^44^, ^47^, ^48^, ^51^, ^52^, ^53^, ^54^, ^55^, ^56^, ^57^, ^59^, ^60^, ^61^, ^63^, ^65^, ^70^, ^74^, ^77^ People also found a consistent point of contact within the institution or external organisation useful.

I personally would have benefited greatly from having some contact with some support from a social worker…just somebody to support us through that time…nursing staff can't do it. They are too busy. It's not to say that they are not very good, they are, but they can't give the support that I felt we needed. (Patient, Iedema et al.^36^ ^, p.95^)

Having this support may increase the perceived trustworthiness of the process by demonstrating the desire to engage with and incorporate their views in the justice‐seeking process. A process which seems to favour the individuals/organisations associated with the original harm may not be viewed as trustworthy, which may influence the perception of a fair or unfair outcome for justice‐seekers.

It is also important that opportunities for justice‐seekers to challenge both the process and outcomes following redress and reconciliation are incorporated into the pathway.^18^, ^20^, ^22^, ^25^, ^27^, ^29^, ^30^, ^34^, ^38^, ^42^, ^49^, ^54^, ^55^, ^56^, ^61^, ^63^, ^77^ This may be through opportunities to correct formal accounts of the life‐changing events, or pursuing formal litigation processes through the courts and/or having their views incorporated within the final verdict. The appeals process represented an important opportunity to challenge final decisions they did not agree with, although many people did not find this easy to access. Appealing could cause unanticipated delays in achieving resolution which, alongside perceived bias in favour of those perceived to have done the harm, can increase the emotional trauma experienced and reduce trust in the process and final outcomes.

After five years of trials, I have two years to ask for a retrial. I don't even know if my retrial demand will go through, I don't know if I can go through another trial if my demand is accepted. (Patient, Pyo et al.^63^ ^, p.6^)

Justice‐seekers highlighted the need for people to be independent from the institution where the harm has occurred, or of the redress‐reconciliation process itself, to contribute to the process.^25^, ^29^, ^32^, ^34^, ^36^, ^38^, ^39^, ^47^, ^51^, ^55^, ^59^, ^61^, ^78^ This objective input supports the provision of information and emotional support to justice‐seekers and oversight of the ongoing investigation, mitigating perceptions of bias. However, mechanisms need to be in place to ensure that these individuals are provided with the correct information and support to enable them to fulfil their role without being seen to compromise the justice process or its outcomes.

### 4.2 What does a fair process feel like?

#### 4.2.1 Theme 4: Restorative justice

The redress‐reconciliation process needs to embody the principles of respect, empathy and good communication, and acknowledge justice‐seekers as equal participants.^18^, ^22^, ^23^, ^25^, ^27^, ^29^, ^30^, ^32^, ^36^, ^43^, ^44^, ^47^, ^49^, ^50^, ^51^, ^52^, ^53^, ^54^, ^55^, ^56^, ^58^, ^59^, ^60^, ^61^, ^65^, ^68^, ^70^, ^71^, ^74^, ^75^, ^76^, ^77^, ^78^ This helps humanise a process that, if too overly focused on its procedural elements, risks being experienced as emotionally harmful, bureaucratic and insensitive to the needs of those who have experienced harm. Embracing these principles may improve the relationship between people who have been harmed and other stakeholders involved in the process, creating opportunities to resolve misunderstandings or inaccuracies and validate the pain experienced. This may reduce feelings of isolation, overwhelm and anger and increase perceptions of fairness of the process and its outcomes.

…she was being like a human being, a women who's a mother herself and she kind of slightly stepped back from her professional role and just spoke to you like an adult…it made us feel good because we knew she cared. (Parent whose baby died, McQueen et al.^76^ ^, p.6^)

Within the context of a humanising process, the process of redress‐reconciliation can support individuals to transition from a position where they are overwhelmed by the trauma they have experienced to a position of acceptance and being able to move on with other areas of their life.^18^, ^19^, ^20^, ^22^, ^25^, ^27^, ^29^, ^30^, ^32^, ^34^, ^35^, ^36^, ^38^, ^39^, ^42^, ^43^, ^44^, ^47^, ^49^, ^50^, ^51^, ^52^, ^53^, ^54^, ^55^, ^57^, ^58^, ^59^, ^60^, ^61^, ^63^, ^64^, ^65^, ^68^, ^70^, ^72^, ^75^, ^76^, ^78^ The procedurally orientated nature of the redress‐reconciliation processes (documented in Themes 1–3) can overlook the emotional needs of justice‐seekers. Developing a shared narrative could provide those who have experienced harm with the opportunity to have their say and receive validation of the hurt they have experienced by those perceived as responsible for the harm.

It's absolutely, fundamentally, about being heard and being able to look the health professionals in their eyes, tell your story, and for them to look you in the eyes, and actually register. (Patient, Moore et al.^60^ ^, p.791^)

This validation, alongside space to express and process emotions, can be cathartic. It allows justice‐seekers to integrate fractured information from multiple sources to construct a complete narrative regarding what happened and for this to be reflected in the public account.

It wasn't until I got the coronial inquest that the video in my head went away. I don't know if you believe that but anyway it was nearly like dad or someone was trying to tell me something is not right. You have to find what actually happened. (Family member bereaved by work accident, Ngo et al.^61^ ^, p.456^)

Justice‐seekers appreciated opportunities for involvement in the redress‐reconciliation system through pursuing accountability from individuals and/or organisations and identifying learning points going forward. This action can help give meaning to the harm they have experienced and provide an end‐point to the narrative documenting their experiences.

Before March I blamed the hospital, I blamed myself, I blamed everybody. Like, the guilt was just so raw with me. My own guilt and the guilt that I'd let my son down, and the blame that I needed to pass on to the hospital, and all of that. Since the Open Disclosure I know for a fact that there has been measures put in place so that this doesn't happen again…The Open Disclosure for me itself actually lifted a great weight off my shoulder. I didn't feel like it was about guilt anymore. It was about acceptance. This happened which shouldn't have happened but it did and I have to accept that and move on. (Family member—Open Disclosure process, Iedema et al.^36^ ^, p.115^)

We propose that the combination of developing a cathartic narrative and opportunities to participate in therapeutic action can help some individuals process their trauma and move through to a place of acceptance and feel able to close this chapter of their lives.

## 5 DISCUSSION

We synthesised 41 primary qualitative studies (47 papers) exploring the experiences of those seeking justice following a life‐changing event. Thirty‐one of these studies focused on patients and/or their families following medically life‐changing events.^19^, ^23^, ^31^, ^32^, ^34^, ^35^, ^36^, ^43^, ^44^, ^47^, ^48^, ^49^, ^50^, ^51^, ^52^, ^53^, ^54^, ^55^, ^56^, ^57^, ^58^, ^59^, ^60^, ^63^, ^65^, ^68^, ^69^, ^70^, ^71^, ^72^, ^74^, ^75^, ^76^, ^77^, ^78^

Three themes, ‘Transparency’, ‘Person‐centred’ and ‘Trustworthy’, represent the procedural elements of redress‐reconciliation required to support a fair and objective process. The elements within these three themes are interdependent. For example, if the process is conducted using a person‐centred approach, this will likely increase the transparency and trustworthiness of the process. If the redress‐reconciliation process is consistent with the procedural elements identified by this synthesis, it may support the development of a supportive, empathic relationship between justice‐seekers and individuals seen as responsible for the harm. This relationship may support justice‐seekers to develop a coherent narrative about the trauma they have experienced. This presents the opportunity to tell their own story and receive acknowledgement for the hurt they have experienced. During this process, justice‐seekers can be supported to take part in actions which give meaning to their loss. The combination of experiencing a humanising process, developing a cathartic narrative and participating in meaningful action provides the foundation for the final theme, ‘Restorative justice’, which encapsulates how a fair process feels to those who have experienced a life‐changing event. It is within the context of the humanising and cathartic relationship between these stakeholders that the procedural elements of the redress and reconciliation process can be worked through, the harm and the impact on the individual can be explored, meaningful outcomes agreed upon and the emotional impact diffused, as people accept what has happened and learn how to incorporate the consequences of the event into their lives. Thus, we propose that a fair process is dependent on both its procedural elements and the quality of the relationship developed between the different stakeholders.

Content from the ‘Transparency’, ‘Person‐centred’ and ‘Trustworthy' themes aligns with the concepts of publicity, relevance and legitimacy proposed by Daniels and Sabin.^15^, ^16^, ^17^ The ‘Trustworthy’ theme within this review also incorporates ideas related to the opportunity to appeal and enforcement conditions also proposed by Daniels et al.^15^, ^16^, ^17^ However, Daniels and Sabin indicate that the involvement of members of the public within the redress‐reconciliation process is not necessary for it to be procedurally fair. Thus, our final theme, ‘Restorative Justice’, is not represented by their work and may also reflect that their work focused on resource allocation rather than individual trauma. Instead, our fourth theme draws upon the restorative justice literature, which emphasises the humanisation of all parties involved with the redress‐reconciliation process, by establishing respectful relationships which encourage listening and emotional expression.^79^ We propose that encouraging these types of relationships can lead to a reduction in anger and resentment, reducing the desire for revenge and retribution on the part of those seeking redress‐reconciliation.

Another concept represented extensively within the framework synthesis was the need for an apology, which itself should encompass expressions of remorse, accountability and assurances that the event will not happen again. The importance of taking accountability, and the limits of this, is recognised within Open Disclosure policies which informed health policy reform across several countries, including the United Kingdom.^65^, ^78^ This was intended to prevent the cycle of avoidance and defensiveness by professionals which can prevent learning from occurring and increase legal action as patients/families seek accountability.^78^ However, healthcare systems are continually evolving, and there are many different factors influencing the care of any individual patient with a variety of different stakeholders working according to different sets of regulations and guidelines. Thus, implementing and maintaining change and identifying patients to whom this change may be relevant can be difficult.^80^

### 5.1 Strengths and limitations

This evidence synthesis was conducted in accordance with the systematic review methodology and reporting guidelines. To ensure that the review was delivered within the agreed time period, we were unable to include all eligible studies in the framework synthesis. However, we ensured that the synthesis prioritised studies reflecting experiences of justice‐seeking following medically adverse events. Our purposive sampling approach helped us incorporate learning from outside the medical field. While papers not included in the synthesis may have contributed additional nuance, it is unlikely the final four themes would be altered. We would welcome the opportunity to further share our work with people with experiences of life‐changing events and incorporate their feedback.

While most subthemes were supported by a robust quantity of primary evidence, this was not the case for all. Most studies supporting this subtheme and ‘Mechanisms for challenge and dispute resolution’ were supported by studies where the life‐changing event experienced was non‐medical. This reflects our purposive sampling strategy, and whilst the learning from these studies may be useful, the extent to which these findings can be generalised to a medical context should be interpreted with caution.

Most primary studies included in this review did not contain information which allowed us to explore how structural determinants, such as the ethnicity and gender of justice‐seekers, may have influenced the perceived fairness of the process.

### 5.2 Implications for policy, practice and future research

Our findings could be used to support professionals involved with the redress‐reconciliation system to establish if those seeking redress‐reconciliation following both recent and historical medically life‐changing events have experienced a fair process. Our findings may support patients and/or their families by informing them what to expect in terms of a fair process, which may help them articulate their needs at different stages throughout their journey.

The findings of this review could be shared with individuals who have experienced medical harm, or the groups/organisations representing them, to establish if the findings of this review reflect their experiences and needs. Qualitative primary research could explore the experiences and needs of this group, with particular emphasis on the need for a clear rationale for decisions made and their views on the resources available to support them to challenge findings/processes or resolve disputes. Emphasis should be placed on seeking the views of individuals from minority ethnic groups who may find it harder to access a fair redress‐reconciliation process.

Primary research evaluating the extent to which existing redress‐reconciliation structures and processes utilised within healthcare systems reflect the components of the fair process outlined above could provide insight into whether current processes are perceived as fair. Some of the potential changes identified may be challenging to incorporate into the procedure‐based systems used within healthcare and other organisations supporting redress‐reconciliation processes. Further research on how to implement any proposed changes would also be required.

### 5.3 Conclusions

This report highlights key features of redress‐reconciliation to consider ensuring the process and outcomes are experienced as fair. The nature of these findings considers the procedural aspects of a fair process and the context in which these need to occur in order that fairness can be achieved. Our findings may be used in relation to processes to investigate recent patient safety events as well as those where the life‐changing events are historical.

## AUTHOR CONTRIBUTIONS

Liz Shaw is the lead author of the review. Led development of the protocol and all stages of conducting the systematic review, including screening, data extraction, quality appraisal and synthesis of the data and write‐up of the paper. Also led stakeholder and patient and public involvement. Hassanat Lawal supported screening, data extraction, quality appraisal and synthesis of the data and writing of the paper. Supported patient and public involvement. Simon Briscoe contributed towards developing the review protocol and designed and implemented the search strategy for the review. Supported screening. Read and commented on draft versions of the paper. Ruth Garside supported the development of the review protocol and preliminary synthesis. Read and commented on draft papers. Jo Thompson Coon supported the development of review protocol and preliminary synthesis. Read and commented on draft papers. Morwenna Rogers supported the development of a search strategy. Read and commented on draft papers. G. J. Melendez‐Torres is the guarantor of this review. Supported development of the protocol and provided oversight of methods and contributed to developing synthesis. Supported stakeholder engagement and read and commented on draft papers.

## CONFLICT OF INTEREST STATEMENT

The authors declare no conflict of interest.

## Data Availability

Data are available on request from the authors. The data that support the findings of this study are available from the corresponding author upon reasonable request.

## APPENDIX A

See Table A1

**Table A1:** Stakeholder involvement with review process.

Stage of review	Stakeholder, mode of contact	Influence on review process
Protocol development	Three representatives from DHSC—remote meeting, email.	Defining our research question. Developing the project protocol, specifically enabling us to identify key populations and the phenomenon of interest. Finalising search terms for bibliographic database searches.
Screening	Two representatives from DHSC—remote meeting, email.	Providing clarification on review inclusion criteria, particularly where service or professional context is relevant to healthcare context or determining if the context of the procedural justice system amenable to the UK setting. Reviewing the list of included reviews following full‐text screening. Providing feedback on studies to include via purposive sampling.
Synthesis/presentation of findings	Three representatives from DHSC—remote meeting. Two people with lived experience of medically adverse events.	Providing feedback on preliminary findings. Ensuring our findings are accessible to our intended audience Feedback on findings. Develop implications for future research and practice.

Abbreviation: DHSC, Department of Health and Social Care.

## APPENDIX B EXAMPLE SEARCH STRATEGY

Bibliographic databases

Database: MEDLINE

Host: Ovid

Issue: 1946 to February 17, 2022

Date Searched: 25/2/2022

Searcher: MR

Hits: 1293

Strategy:

1. Malpractice/
2. medical errors/
3. diagnostic errors/
4. missed diagnosis/
5. medication errors/
6. inappropriate prescribing/
7. medication reconciliation/
8. near miss, healthcare/
9. (medical adj1 (accident* or error* or injur*)).ti,ab.
10. (Incident or incidents).ti,ab.
11. negligen*.ti,ab.
12. adverse event*.ti,ab.
13. patient safety.ti,ab.
14. ((sudden or unexpected) adj2 (death* or mortalit*)).ti,ab.
15. (death* adj5 custody).ti,ab.
16. malpractice.ti,ab.
17. (homicide* or manslaughter).ti,ab.
18. suicide*.ti,ab.
19. 1 or 2 or 3 or 4 or 5 or 6 or 7 or 8 or 9 or 10 or 11 or 12 or 13 or 14 or 15 or 16 or 17 or 18
20. Patient Advocacy/
21. ((death or mortality) adj1 review*).ti,ab.
22. inquest*.ti,ab.
23. postmort?m*.ti,ab.
24. (medicolegal or medico legal).ti,ab.
25. incident investigation*.ti,ab.
26. complaint*.ti,ab.
27. litigation*.ti,ab.
28. (ombudsman or ombudsmen).ti,ab.
29. settlement*.ti,ab.
30. (malpractice adj2 claim).ti,ab.
31. mediation.ti,ab.
32. advocacy. ti,ab.
33. (accountable or accountability).ti,ab.
34. (legitimate or legitimacy).ti,ab.
35. (disclosure adj2 (policy or policies or process*)).ti,ab.
36. “child protection”.ti,ab.
37. or/20‐36
38. “Compensation and Redress”/
39. resolution.ti,ab.
40. reconciliation.ti,ab.
41. disclosure.ti,ab.
42. justice.ti,ab.
43. (accepting or acceptance or anger or distress* or distrust* or feelings or frustration or grief or ignored or mistrust* or stress or stressful or traumati* or trust*).ti,ab.
44. bereav*.ti,ab.
45. fairness.ti,ab.
46. responsibility.ti,ab.
47. support.ti,ab.
48. (satisfaction or satisfied or dissatisfaction or dissatisfied).ti,ab.
49. apolog*.ti,ab.
50. closure.ti,ab.
51. or/38‐50
52. qualitative research/
53. qualitative*.ti,ab.
54. (experience or experiences or perspective* or phenomenolog*).ti,ab.
55. interview*.ti,ab.
56. (survey* or “focus group*“).ti,ab.
57. or/52‐56
58. 19 and 37 and 51 and 57

## APPENDIX C

See Table C1

**Table C1:** Inclusion criteria.

	Associated inclusion criteria
Population	*Include*: Individuals who have experienced a life‐changing event. These individuals may include the person who has experienced the event or family/carers seeking justice on behalf of the person who experienced the event. *Exclude*: Staff about whom the complaint has been made or who are working within the redress/reconciliation system.
Phenomenon of interest	*Include*: Experiences and/or views of redress and reconciliation processes following a life‐changing event. Experiences and/or views on what is perceived to be a fair/unfair outcome following a life‐changing event. *Exclude*: Experiences of justice‐seeking processes where this is against individual members of staff, not the system/facility as a whole. Experiences and/or views of redress and reconciliation processes following life‐changing events where the primary outcome/phenomenon of interest is the impact on grief and/or bereavement.
Context	Redress/reconciliation processes occurring within the following settings: Health or social care systems following life‐changing events (e.g., adverse patient safety events); child protection or sudden child death investigations; homicide reviews and restorative justice processes within a criminal context; any other service or professional context identified by our searches where findings are amenable to importing into the health care context.
Geographical region	High‐income countries as defined by the World Bank list. This was to ensure countries represented by the review findings had similar procedural justice systems and be more likely to be applicable to a UK setting.
Date of publication	No restriction.
Study design	Any study design collecting the experiences/views of individuals defined within the “Population” section above.
Key definitions	*Redress*: To make amends for or give payment for a wrong which has been done.^81^ *Reconciliation*: A process which presents the opportunity for the sides of a conflict to express concerns about a past event, have these concerns validated and seek to move beyond these in a renewed relationship. The reconciliation process also incorporates opportunities for redress.^82^ *Life‐changing event*: Draws upon the definition of “serious adverse event” used within medical settings, which is any “untoward medical occurrence(s) that at any dose results in death, hospitalisation or prolongation of existing hospitalisation, persistent or significant disability/incapacity or a congenital anomaly or birth defect.”^83^ However, this definition can also be applied to events which occur outside of medical settings, as listed within the “Context” section above

Abbreviations: PICo, population, phenomenon of interest, context; UK, United Kingdom.

## APPENDIX D

See Table D1

**Table D1:** Stages at which prioritised articles are added to framework synthesis.

Stage of framework synthesis	Description	Supporting papers, first author (date)
Stage 1: Papers from the medical field with the highest quantity of information relevant to our research questions. This process meant that the medical studies with the greatest quantity of conceptually rich data had the greatest influence in shaping the developing framework, ensuring it best reflects the findings of papers most relevant to our review question: *N* = 17.	Coding of first‐ and second‐order data medical studies containing the highest quantity of data relevant to our research question from across different stages of the justice‐seeing pathway (L. S.). Data mapped onto four conditions identified by Daniels and Sabin (1997; 1998; 2000),^15^, ^16^, ^17^ which formed the basis of early subthemes. Relevant data are not easily mapped onto the four conditions listed separately (L. S.). Data extracted from each study were checked to ensure that all data relevant to our research questions had been captured (H. L.). Inductive coding of the content contained within each of the subthemes to group similar concepts together. This involved moving groups of codes across different subthemes and helped to ensure that the developing subthemes remained conceptually distinct from one another (L. S.). Placement of descriptive codes within each subtheme discussed between two reviewers (L. S., H. L.), before receiving feedback on the developing framework from members of the wider team (G. J. M., R. G., J. T. C., S. B.).	Bakhbakhi (2017),^48^ Chiu (2010),^50^ IPSOS (2016)^55^ Duclos (2005),^51^ Hagensen (2018)^53^ Iedema (2007, 2011, 2012a),^34^, ^35^, ^36^ Kim (2021),^70^ Martin (2021),^57^ McQueen (2021),^76^ Melville (2012),^77^ Moore (2017a, 2017b),^59^, ^60^ Myren (2021),^44^ Pyo (2019),^63^ Sorensen (2010).^65^
Stage 2: Remaining papers from the medical field with a medium or low quantity of information relevant to our research questions: *N* = 18.	First‐ and second‐order construct data from remaining medical studies coded using the descriptive codes from the revised framework. Additional codes were added as required and applied to previously coded studies in an iterative process (L. S.). Key concepts from this second group of papers were used to revise how descriptive codes were grouped, resulting in the generation of a new theme containing two new subthemes. Names and positions of other existing subthemes to reflect their content (L. S.). Names of existing themes were checked to ensure that they accurately reflected the content and the relationship between the subthemes within them (L. S.). The wider team (G. J. M., R. G., T. C., S. B.) provided input regarding theme labels within the revised framework and the proposed relationship between the different themes and subthemes, which were incorporated into the synthesis (L. S.).	Fisher (2016),^68^ Gallhager (2009),^52^ Loren (2021),^71^ Kent (2008),^56^ Mazor (2010, 2012, 2013),^43^, ^58^, ^72^ Piper (2014),^74^ Wiig (2021),^47^ Boumann (2018),^49^ Butler (2019),^19^ Kamin‐Friedman (2021),^23^ Hernan (2014),^69^ Hannawa (2017),^32^ Hovey (2014),^75^ Iedema (2012b),^54^ Etchegary (2014),^31^ Ocloo (2010).^78^
Stage 3: Papers from nonmedical fields which contained data relevant to weaker subthemes “Mechanism for challenge” and “Rationale for decisions.” Also added papers which spoke to the concept that for some individuals justice is not possible: *N* = 12	Nonmedical studies were examined independently by two reviewers (L. S., H. L.) for data to support weaker subthemes within the framework. Disagreements were resolved through discussion, and these studies added to the framework. Changes in position and names of subthemes to reflect content. Structure of final themes influenced by Restorative Justice literature,^79^ and content of second stage medical studies.	Biddle (2002),^18^ Burns (2006),^27^ Eastwood (1998a, 1998b),^22^, ^29^ Englebrecht (2014),^30^ Maderia (2008, 2010),^38^, ^39^ Matthews (2012),^42^ Chapple (2012),^20^ Ngo (2021),^61^ Saco (2018)^64^ Shaw (2007).^25^

Abbreviation: *N*, number.

## APPENDIX E DEVELOPMENT OF THE BEST‐FIT FRAMEWORK

See Tables E1, E2, E3

**Table E1:** Best‐fit framework—Version 1.

V1: Themes Based on Daniels and Sabin	Theme 1: Transparency/Publicity condition	Theme 2: Relevance condition	Theme 3: Legitimacy of process	Theme 4: Opportunity to appeal rationale	Theme 5: Enforcement condition	Theme 6: Other
Subthemes	That an error has occurred: Disclosure of relevant information (allow individuals to make choices regarding care/clinicians/treatment).	Shared ‘rules of the game’: An agreed process for achieving justice. An agreed goal/outcome to be achieved and consideration of relevant points to all parties.	Informed choice of options available.	Mechanism for challenge and dispute resolution regarding limit‐setting decisions, opportunity for revising decisions in light of further evidence or arguments.	Voluntary or public regulation of the justice‐seeking process.	First/second‐order construct data which appears relevant to research question but does not fit in current version of framework.
	Information required publicly available.	Does outcome/plan match needs of justice‐seekers?	Ongoing support for justice‐seekers (health and legal).			
	Consideration of things which could constrain decision‐making: Government policy, institutional rules.	Explicit plan: What is going to happen as a result of outcome	Who is accountable?			
	Of rationales for decisions made during the justice‐seeking process: What decision was reached, how and why?	Use of a reasonable and consistent process to determine justice?				

**Table E2:** Best‐fit framework—Version 2.

V.2 Addition of descriptive codes. First changes made regarding placement of content within and across themes/subthemes	Theme 1: Transparency/publicity condition	Theme 2: Relevance condition	Theme 3: Legitimacy of process	Theme 4: Opportunity to appeal rationale	Theme 5: Enforcement condition	Theme 6: Other
Subthemes	02 Information required publicly available.	05 Shared rules of the game—An agreed process for achieving justice. An agreed goal, outcome to be achieved and consideration of relevant points to all parties.	08 Use of a reasonable and consistent process to determine justice.	12 Mechanism for challenge and dispute resolution regarding limit‐setting decisions, opportunity for revising decisions in light of further evidence or arguments.	13 Voluntary or public regulation of the justice‐seeking process.	First/second‐order construct data which appears relevant to research question but does not fit in current version of framework.
	03 Consideration of things which could constrain decision‐making—Government policy, institutional rules.	06 Does outcome, plan match needs of justice‐seekers.	09 Informed choice of options available.			
	04 Of rationales for decisions made during the justice‐seeking process—What decision was reached, how and why.	07 Explicit plan—What is going to happen as a result of the outcome.	10 Ongoing support for justice‐seekers (health and legal).			
			11 Who is accountable.			

*Note*: Yellow highlight—Change to framework since previous version.

**Table E3:** Best‐fit framework—Version 3.

**V.3 Further iterative coding of information to descriptive codes. Changes in position and names of subthemes to reflect content. Creation of final theme ‘Restorative Justice’**	**001 Need for Transparency**	**002 Relevance of process to justice‐seeker**	**003 Trustworthy**	**004 Restorative Justice**
Subthemes	01 An account.	05 Shared rules: An agreed process for achieving justice. An agreed goal, outcome to be achieved and consideration of relevant points to all parties.	07 Use of a reasonable and consistent process to determine justice.	12 Humanising process.
	02 Information required publicly available.	06 Does the outcome match the needs of justice‐seekers.	08 Informed choice of options available.	13 Closing a chapter (encompasses cathartic narrative and meaningful action).
	03 Consideration of things which could influence the justice‐seeking process.		09 Ongoing support for justice‐seekers (health and legal).	
	04 Of rationales for decisions made during the justice‐seeking process—What decision was reached, how, why.		10 Mechanism for challenge and dispute resolution regarding limit‐setting decisions, opportunity for revising decisions in light of further evidence or arguments. 11 Objective input.	

*Note*: Yellow highlight—Change to framework since previous version.

## APPENDIX F

See Table F1

**Table F1:** Quality appraisal scores.

**Study author (date)**	**1‐Research question clear?**	**2‐Theoretic/ideological perspective explicit?**	**3‐Has it influenced study design, methods, findings?**	**4‐Study design appropriate to question?**	**5‐Setting adequately described?**	**6‐Sample drawn from appropriate population?**	**7‐Data collection adequately described?**	**8‐Data collection rigorous to ensure confidence in findings?**	**9‐Data analysis rigorous to ensure confidence in findings?**	**10‐Findings substantiated by data?**	**11‐Consideration given to limitations that may affect results?**	**12‐Claims to generalisability follow from data?**	**13‐Ethics addressed confidentiality respected?**	**14‐Justice‐seeking process clearly described?**
Bakhbakhi (2017)^48^	Y	N	CT	Y	Y	Y	Y	Y	Y	Y	Y	Y	Y	N
Biddle (2003)^18^	Y	N	CT	Y	N	Y	Y	Y	Y	Y	Y	Y	Y	N
Bouwman (2018)^49^	Y	N	CT	Y	N	Y	Y	Y	Y	Y	Y	Y	Y	Y
Burns (2006)^27^	Y	Y	N	Y	Y	Y	Y	Y	Y	Y	Y	Y	Y	Y
Butler (2019)^19^	Y	Y	Y	Y	N	Y	Y	Y	Y	N	Y	Y	Y	Y
Chapple (2012)^20^	Y	N	CT	Y	Y	Y	Y	Y	Y	Y	Y	Y	Y	Y
Chiu (2010)^50^	Y	Y	Y	Y	N	Y	Y	CT	Y	Y	Y	Y	Y	N
Duclos (2005)^51^	Y	N	CT	Y	N	Y	Y	Y	Y	Y	Y	Y	Y	Y
Eastwood (1998a)^22^	Y	N	CT	Y	N	Y	Y	Y	N	Y	Y	Y	CT	N
Eastwood (1998b)^29^	Y	Y	Y	Y	Y	Y	Y	Y	Y	Y	Y	Y	Y	N
Englebrecht (2014)^30^	Y	N	CT	Y	Y	Y	Y	Y	N	Y	Y	Y	N	N
Etchegaray (2014)^31^	Y	N	CT	Y	Y	Y	Y	Y	Y	Y	Y	Y	Y	Y
Fisher (2016)^68^	Y	N	CT	Y	Y	Y	Y	CT	Y	Y	Y	Y	Y	N
Gallagher (2009)^52^	N	N	CT	CT	CT	Y	N	CT	CT	Y	CT	CT	CT	N
Hagensen (2018)^53^	Y	Y	Y	Y	Y	Y	Y	Y	Y	Y	Y	Y	Y	N
Hannawa (2017)^32^	Y	Y	Y	Y	Y	Y	Y	Y	Y	CT	Y	Y	Y	Y
Hernan (2014)^69^	Y	N	CT	Y	Y	Y	Y	Y	Y	Y	Y	Y	Y	N
Hovey (2014)^75^	Y	Y	Y	Y	N	Y	Y	Y	Y	Y	N	Y	CT	N
Iedema (2007)^36^	Y	N	CT	Y	N	Y	Y	Y	Y	Y	Y	Y	Y	N
Iedema (2011)^35^	Y	N	CT	Y	Y	Y	Y	Y	Y	Y	Y	Y	Y	N
Iedema (2012a)^34^	Y	N	CT	Y	Y	Y	Y	Y	Y	Y	Y	Y	Y	N
Iedema (2012b)^54^	N	N	CT	CT	Y	Y	Y	CT	Y	Y	Y	Y	Y	Y
IPSOS MORI (2016)^55^	Y	N	CT	Y	N	Y	N	CT	CT	Y	Y	Y	CT	N
Kamin‐Friedman (2021)^23^	Y	N	CT	Y	N	Y	Y	Y	CT	Y	N	Y	Y	Y
Kent (2008)^56^	Y	Y	Y	Y	N	CT	N	CT	CT	Y	Y	Y	CT	Y
Kim (2021)^70^	Y	Y	Y	Y	Y	Y	Y	Y	Y	Y	Y	Y	Y	N
Loren (2021)^71^	Y	N	CT	Y	N	Y	Y	Y	Y	Y	Y	Y	Y	N
Maderia (2008)^38^	Y	Y	Y	Y	Y	Y	N	CT	CT	Y	N	Y	N	N
Maderia (2010)^39^	Y	Y	Y	Y	Y	Y	N	CT	CT	Y	N	Y	N	N
Martin (2021)^57^	Y	Y	Y	Y	N	Y	Y	CT	Y	Y	Y	Y	Y	N
Matthews (2012)^42^	Y	N	CT	Y	N	Y	Y	Y	CT	Y	Y	Y	N	N
Mazor (2010)^43^	Y	N	CT	Y	N	Y	Y	Y	Y	Y	Y	Y	Y	N
Mazor (2012)^72^	Y	N	CT	Y	Y	Y	Y	Y	Y	N	Y	Y	Y	N
Mazor (2013)^58^	Y	N	CT	Y	Y	Y	Y	Y	Y	Y	Y	Y	Y	N
McQueen (2021)^76^	Y	N	CT	Y	Y	Y	Y	Y	Y	Y	Y	Y	Y	Y
Melville (2012)^77^	Y	N	CT	Y	N	Y	N	Y	N	Y	Y	Y	Y	Y
Moore (2017a)^59^	Y	N	CT	Y	Y	Y	Y	Y	Y	Y	Y	Y	Y	Y
Moore (2017b)^60^	Y	N	CT	Y	Y	Y	Y	Y	Y	Y	Y	Y	Y	Y
Myren (2021)^44^	Y	N	CT	Y	Y	Y	Y	Y	Y	Y	Y	Y	Y	Y
Ngo (2021)^61^	Y	N	CT	Y	N	Y	Y	Y	Y	Y	Y	Y	Y	Y
Ocloo (2010)^78^	N	Y	Y	CT	N	Y	N	CT	CT	N	N	Y	Y	N
Piper (2014)^74^	Y	N	CT	Y	N	CT	Y	Y	Y	Y	Y	Y	Y	N
Pyo (2019)^63^	Y	N	CT	N	Y	Y	Y	Y	Y	Y	Y	Y	Y	Y
Saco (2018)^64^	Y	N	CT	Y	N	Y	Y	Y	Y	Y	Y	Y	N	N
Shaw (2007)^25^	N	N	CT	Y	N	Y	Y	CT	CT	Y	N	Y	N	N
Sorensen (2010)^65^	Y	N	CT	Y	N	Y	Y	Y	Y	Y	Y	Y	CT	N
Wiig (2021)^47^	Y	N	CT	Y	Y	Y	Y	Y	Y	Y	Y	Y	Y	N

Abbreviations: CT, cannot tell; N, no; Y, yes.

## APPENDIX G

See Table G1

**Table G1:** Articles supporting themes.

Study author (date)	Theme 1: Transparency				Theme 2: Person‐centred		Theme 3: Trustworthy				Theme 4: Restorative justice	
	1.1 An account	1.2 Information publicly available	1.3 Consideration of systemic factors	1.4 Clear rationale for decisions	2.1 Shared rules	2.2 Meaningful outcomes	3.1 Consistent process	3.2 Ongoing support	3.3 Mechanisms for challenge or dispute resolution	3.4 Objective input	4.1 Humanising Process	4.2 Closing a chapter
Bakhbakhi (2017)^48^		x			x	x	x	x				
Biddle (2003)^18^	x	x			x	x	x		x		x	x
Bouwman (2018)^49^	x	x		x	x		x		x		x	x
Burns (2006)^27^	x	x	x		x	x	x		x		x	x
Butler (2019)^19^												x
Chapple (2012)^20^	x					x			x			x
Chiu (2010)^50^	x	x			x	x	x				x	x
Duclos (2005)^51^	x	x			x	x	x	x		x	x	x
Eastwood (1998a)^22^		x		x	x		x		x		x	x
Eastwood (1998b)^29^		x	x	x	x	x	x	x	x	x	x	x
Englebrecht (2014)^30^			x	x	x	x	x	x	x		x	x
Etchegaray (2014)^31^					x	x	x					
Fisher (2016)^68^	x	x				x	x				x	x
Gallagher (2009)^52^	x				x	x	x	x			x	x
Hagensen (2018)^53^	x	x				x	x	x			x	x
Hannawa (2017)^32^	x	x			x	x	x	x		x	x	x
Hernan (2014)^69^	x					x						
Hovey (2014)^75^	x	x	x		x	x	x				x	x
Iedema (2007)^36^	x	x			x	x	x	x		x	x	x
Iedema (2011)^35^	x	x			x	x	x	x				x
Iedema (2012a)^34^		x				x	x		x			x
Iedema (2012b)^54^	x	x			x	x	x	x	x		x	x
IPSOS MORI (2016)^55^	x	x	x	x	x	x	x	x	x	x	x	x
Kamin‐Friedman (2021)^23^			x					x			x	
Kent (2008)^56^		x	x			x	x	x	x		x	
Kim (2021)^70^	x	x	x		x	x		x				x
Loren (2021)^71^	x	x				x					x	
Maderia (2008)^38^	x					x		x	x	x		x
Maderia (2010)^39^	x	x		x		x				x		x
Martin (2021)^57^			x		x	x	x	x				x
Matthews (2012)^42^	x	x	x			x	x	x	x			x
Mazor (2010)^43^	x	x				x		x			x	x
Mazor (2012)^72^	x					x						x
Mazor (2013)^58^	x				x	x	x				x	x
McQueen (2021)^76^	x	x	x		x	x	x				x	x
Melville (2012)^77^	x	x	x	x	x	x	x	x	x		x	
Moore (2017a)^59^	x	x	x		x	x	x	x		x	x	x
Moore (2017b)^60^	x	x	x		x	x	x	x			x	x
Myren (2021)^44^	x	x				x	x	x			x	x
Ngo (2021)^61^	x	x				x	x		x	x	x	x
Ocloo (2010)^78^	x	x	x			x				x	x	x
Piper (2014)^74^	x					x	x	x			x	
Pyo (2019)^63^		x		x		x		x	x			x
Saco (2018)^64^						x						x
Shaw (2007)^25^	x	x	x		x	x	x	x	x	x	x	x
Sorensen (2010)^65^		x			x	x	x	x			x	x
Wiig (2021)^47^		x				x	x	x			x	x
